# Numerical Modeling for the Prediction of Microstructure and Mechanical Properties of Quenched Automotive Steel Pieces

**DOI:** 10.3390/ma16114111

**Published:** 2023-05-31

**Authors:** Carlos Coroas, Iván Viéitez, Elena Martín, Manuel Román

**Affiliations:** 1 Departamento de Matemática Aplicada II, Escuela de Ingeniería de Telecomunicación, Campus Marcosende, Universidade de Vigo, 36310 Vigo, Spain; 2 Instituto de Física y Ciencias Aeroespaciales, Campus Ourense IFCAE, Universidade de Vigo, 32004 Ourense, Spain; 3 CIE—Galfor Company, P.I. San Cibrao das Viñas, Calle 2, 3, 32901 Ourense, Spain

**Keywords:** numerical simulation, heat treatment, quenching in liquids, microstructure and distortion prediction, phase transformation

## Abstract

In this work, we present an efficient numerical tool for the prediction of the final microstructure, mechanical properties, and distortions of automotive steel spindles subjected to quenching processes by immersion in liquid tanks. The complete model, which consists of a two-way coupled thermal–metallurgical model and a subsequent (one-way coupled) mechanical model, was numerically implemented using finite element methods. The thermal model includes a novel generalized solid-to-liquid heat transfer model that depends explicitly on the piece’s characteristic size, the physical properties of the quenching fluid, and quenching process parameters. The resulting numerical tool is experimentally validated by comparison with the final microstructure and hardness distributions obtained on automotive spindles subjected to two different industrial quenching processes: (i) a batch-type quenching process with a soaking air-furnace stage prior to the quenching, and (ii) a direct quenching process where the pieces are submerged directly in the liquid just after forging. The complete model retains accurately, at a reduced computational cost, the main features of the different heat transfer mechanisms, with deviations in the temperature evolution and final microstructure lower than 7.5% and 12%, respectively. In the framework of the increasing relevance of digital twins in industry, this model is a useful tool not only to predict the final properties of quenched industrial pieces but also to redesign and optimize the quenching process.

## 1. Introduction

In the industrial quenching process, metal pieces at a high temperature (above the austenitization temperature) are immersed in a tank filled with liquid (usually subjected to agitation) to be cooled very rapidly. As an example, the inner temperature of steel pieces drops from temperatures typically in the range of 900–1000 °C to the fluid saturation temperature in less than 5 s. The objective of this heat treatment is to induce metallurgical transformations in the material that generate specific micro-structures, specifically to obtain a martensitic exterior zone and a bainitic interior, which provide desirable mechanical characteristics with great surface hardness, high tensile stresses, and great resistance. At the same time, this rapid cooling induces residual stresses and geometrical distortions in the pieces [1]. The process involves the coupling of different complex physical mechanisms: heat transfer between a dynamical multiphasic fluid problem and the solid piece, a metallurgical transformation problem, and a solid-mechanic stress problem. The piece’s thermal evolution hugely conditions the final properties of the piece, as it is strongly coupled to the material microstructure evolution and the mechanical problem, which finally determine the tensional state of the piece and its deformation (see [2] for a complete review on the coupling and modeling of the different mechanisms).

Modeling the evolution of residual stresses and geometrical distortions of solids has a sound background based on continuous mechanics [3]. The phase transformation problem is also described with models (some of them empirical) accurate enough to be applied to the quenching process [4]. However, the heat transfer problem in the quenching involves the dynamical generation/destruction of vapor bubbles and/or a vapor film of the quenching fluid surrounding the piece in very short time scales [5,6]. Therefore, complete, detailed dynamical two-phase fluid models with heat transfer capable of solving all the phenomena that appear (at different temporal and spatial scales) have not been tackled yet. The implementation of a complete model implies, on the one hand, solving physics problems (such as the generation, departure, and condensation of vapor bubbles) that occur at characteristic lengths of the order of microns and, on the other hand, solving turbulent fluid flow and heat transfer problems up to lengths of the order of the piece size. Only a few attempts at solving the multi-phase problem under very simplified assumptions have been made, most of them not specifically aimed at the analysis of industrial quenching of steel pieces but at the nuclear engineering industry conditions ([7,8,9,10]). Despite the great simplifications introduced by these multi-phase models, the computational effort and time required to be implemented correctly make them ineligible to simulate the quenching of complex industrial pieces.

In the context of industrial quenching modeling of steel pieces, the common approaches used to estimate the heat transfer between the surface piece and the two-phase fluid are two: (i) to approximate or adjust a heat transfer coefficient through experimental testing campaigns; or (ii) to approximate the heat transfer coefficient using adaptations of correlations from literature ([11,12,13,14,15,16,17,18,19,20]). The first approach presents some drawbacks: First, it requires the realization of test campaigns to measure the piece’s temperature evolution. This, in turn, needs the placement of thermocouples inside the industrial pieces with high data sampling (in the range of 100 Hz), which for most of the industrial processes can be very tricky to carry out and, for certain quenching fluids (e.g., oils), cannot be accomplished. Additionally, the subsequent resolution of the inverse heat transfer problem to obtain the heat transfer coefficient (see [21,22] as examples for simplified geometries) can lead to high uncertainties when dealing with complex pieces. Furthermore, once the heat transfer coefficient is obtained, it can only reproduce the quenching problem when subjected to the same quenching conditions (e.g., same piece geometry, position, and composition; piece initial temperature; type of fluid; agitation velocity; and bulk temperature). Consequently, a large number of test campaigns must be carried out. All this usually leads to performing the tests using probes with simplified geometries [23] at lab scale, which differ substantially from the industrial pieces, leading to heat transfer coefficients that are far from those of the industrial quenched pieces. Therefore, the approach of describing the piece/fluid interaction by means of heat transfer correlations is more attractive since it provides more flexibility to adjust the model to different conditions of the quenching process (e.g., piece size, type of quenching fluid, quenching fluid temperature, agitation velocities, etc.).

The objective of this work is to develop a numerical tool to test the suitability (in terms of attaining the appropriate final microstructure and mechanical properties of automotive spindles) of different industrial quenching processes. The tool, based on solving first a (two-way coupled) thermal–metallurgical model and subsequently a mechanical model (one-way coupling), predicts accurately and efficiently (in terms of computational time) the final microstructure distribution, mechanical properties, and distortions of the pieces subjected to different heat treatment conditions. Consequently, the tool can be used to redesign the quenching process. The thermal model is based on the use of different heat transfer correlations, some of which are proposed in this work, to obtain the heat transfer between the fluid and the piece surface for all the different regimes that take place during the cooling. Despite the simplicity of the proposed thermal model, its numerical results constitute a huge improvement in accuracy in relation to the common approaches used to predict the thermal history and, therefore, the final piece of microstructure.

The work is organized as follows: once the industrial quenching problem is presented in Section 2, the complete model is described in Section 3. Firstly, the thermal model is detailed in Section 3.1 and validated with laboratory experimental tests in Section 3.2 to prove its accuracy. Then, the metallurgical and mechanical models are presented in Section 3.3 and Section 3.4, respectively, and the implementation of the complete model is described in Section 3.5. In Section 4, the numerical results and their validation applied to two different quenching processes: (i) the industrial quenching process previously described in Section 2, and (ii) a modified quenching process (Section 4.1) are presented. Finally, conclusions are summarized in Section 5.

## 2. Description of the Industrial Quenching Process

**Figure 1:**
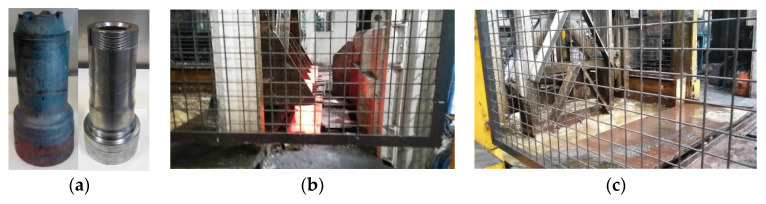
(**a**): Manufactured axle spindles: Aspect of the piece after quenching (left spindle) and a final manufactured spindle (right spindle). (**b**) Images of the manipulation of the spindles after the homogenizing furnace. (**c**) Submersion in the quenching bath. Images provided by CIE GALFOR S.A.

The pieces analyzed in this work are truck axle spindles of 15 kg (whose length and averaged diameter and thickness will be denoted henceforth as L, D, and e, respectively), manufactured by CIE GALFOR (http://www.cieautomotive.com/-/cie-galfor (accessed on 25 May 2023)) (Spain). The specific spindle analyzed in this work is made of low-alloyed steel classified into the F-130 group by the National Spanish Center of Metallurgical Research CENIM (http://www.cenim.csic.es/index.php (accessed on 25 May 2023)) and referenced as F-130DEH. It is a high-resistance steel with a similar composition to 25 M 6 Steel, with a carbon content %C of 0.23. Figure 1a shows the spindle aspect just after the quenching treatment (left figure) and the final aspect of the piece (right figure). These pieces work as joint devices between the truck wheels, the axle, and the suspension system of the vehicles, and are subjected to intense forces, wear, and friction.

As great resistance and strength are needed, a rapid cooling (quenching) process in water is performed in the manufacturing process after the hot forging to ensure the appropriate final martensitic microstructure that provides the mechanical characteristics needed. Rods of the unforged material with 130 mm of diameter are heated until they reach the forging temperature. Afterward, the rods are cut into billets 14 cm long and undergo a minimum of two forging stages with vertical punch dies.

The company manufactures the axle spindles in batches of four and submerges them in a non-pressurized fluid tank, as can be seen in Figure 1b,c. After the forging stage, a natural gas continuous (pusher type) furnace is used to increase and homogenize the piece’s temperature above the material’s austenizing temperature. The homogenization temperature, which depends on the material’s chemical composition and the size of the manufactured pieces, is usually in the range of 850 to $1050^{\;\circ }C$. This heating also reduces the stresses induced on the pieces during the forging process. After the tray is pushed out of the furnace, an automated mechanism elevates the four pieces that hang from a supporting device (Figure 1b) and submerges them in the quenching bath (Figure 1c). This transportation process lasts for 27 s, while the complete immersion of the piece takes 4 s more. The measurement of temperatures at four stages of this process (exit from the homogenization furnace, positioning in the transportation device, beginning of the downward movement, and the instant just before the immersion) was tracked with a pyrometer. The values indicated in Table 1 show that the cooling of the air during transportation is not negligible.

The quenching liquid is water with a residual polymer concentration of less than 1% at 35 °C (308 K). The tank has an agitation system that generates a vertical velocity of 0.34 m/s upstream of the piece tray. The pieces remain in the bath for 378 s until they are removed.

## 3. Numerical Model

The three physics involved in the problem will be solved in the following way: the thermal and microstructure evolution will be solved with a two-way coupling. This is necessary because thermal evolution governs the metallurgical phases while, at the same time, the thermal properties of the piece (thermal conductivity and specific heat) are strongly dependent on the metallurgical phases. Moreover, important latent heat is generated during the evolution of the metallurgical phases. Once the thermal and microstructure evolutions are solved, the mechanical problem will be solved with a one-way coupling. The International System of Units is used to express all constants and variables involved in the following equations.

### 3.1. Thermal Model

The PDE to be solved to obtain the piece temperature field $T\left(\vec{x},t\right)$ in the domain Ω corresponds to the unsteady heat diffusion Equation (1), a heat transfer boundary condition at the fluid-solid interface ∂Ω defined in (2) is used, and the appropriate temperature initial condition is defined by (3):

(1) $$\rho C_{p}\frac{\partial T}{\partial t}-\nabla \cdot \left(k\nabla T\right)=Q\;\;\;\;\;\mathrm{at}\;\;\;\;\Omega$$

(2) $$-k\nabla T\cdot \vec{\eta }=q\left(T_{w}\right)=h_{c}\left(T_{w}-T_{ref}\right)+q_{rad}\;\;\;\mathrm{at}\;\;\;\partial \Omega$$

(3) $$T\left(\vec{x},t=0\right)=T_{0}\left(\vec{x}\right)$$

where ρ, k, and $C_{p}$ are the material density, thermal conductivity, and specific heat, and $\vec{\eta }$ is the local outgoing normal unit vector at the piece surface ∂Ω. $T_{w}$ stands for the piece’s surface temperature. For the steel material, density, in $kg/m^{3}$, is made dependent on temperature as follows:

$$\rho \left(T\right)=7800-0.35\left(T-273K\right)$$

Thermal properties k and $C_{p}$ are defined as functions of the proportion of the phase i, where i can be austenite γ (hot phase) or ferrite f, pearlite p, bainite b, and martensite m (cold phases).

$$\begin{array}{ccc} k\left(T\right) & = & \sum_{i=\gamma ,f,p,b,m}k_{i}\left(T\right)X_{i}\\ C_{p}\left(T\right) & = & \sum_{i=\gamma ,f,p,b,m}C_{p,i}\left(T\right)X_{i} \end{array}$$

$k_{i}$ and $C_{p,i}$ are extracted from [24]. In Equation (1), the term Q represents the heat source associated with the heat released by the metallurgical transformations during the cooling and depends on the transformation rates of each solid phase and its enthalpy of solid phase change $\Delta H_{i}$ ([24]):

$$Q=\rho \left(T\right)\sum_{i}\Delta H_{i}\frac{\partial X_{i}}{\partial t}$$

The thermal boundary condition (2) involves the determination of the heat flux $q\left(T_{w}\right)$, or alternatively, the global heat transfer coefficient h, defined as $q\left(T_{w}\right)=h\left(T_{w}-T_{ref}\right)$, which accounts for all types of heat transfer between the surrounding fluid and the piece. The heat transfer coefficient $h_{c}$ in Equation (2) represents only the convective contribution. 

After the heating in the furnace, two different stages will be considered in this model:

1. A first one, where the pieces are transported from the outlet of the furnace to the quenching bath, being in contact with the surrounding air and the transportation tray.
2. A second one, with the pieces submerged in the fluid.

In turn, this second stage can be divided, depending on the quenching fluid properties and the bath agitation, into the well-known three consecutive regimes [6]: film boiling, nucleate boiling, and single-phase regime. If film boiling appears, a vapor film surrounds the wall, insulating the piece. Thus, heat fluxes are moderate. When a specific temperature is reached, named the Leidenfrost point (LDF), the vapor destabilizes, and a new regime with the generation of vapor bubbles over the wall appears. As the cooling continues, a fully developed boiling appears, with the heat transfer reaching a maximum value, $q_{CHF}$, usually named critical heat flux (CHF). Henceforth, the heat flux decreases as fewer and fewer bubbles are formed at the piece’s surface (what is called partial boiling). Finally, when the production of bubbles stops, only single-phase heat transfer for a liquid takes place.

In this work, we propose a functional surface heat flux $q\left(T_{w}\right)$ that depends, among others, on the characteristic dimensions of the piece:

- Piece length L, diameter D, and thickness e, and on the following quenching parameters:
- Bulk quenching liquid temperature $T_{b}$
- Velocity V of the quenching liquid upstream of the piece
- Thermophysical properties of the quenching fluid: liquid and vapor densities, viscosities, conductivities, and specific heats $\rho_{l}$, $\rho_{v}$, $\mu_{l}$, $\mu_{v}$, $k_{l}$, $k_{v}$, $C_{p,l}$, $C_{p,v}$, saturation temperature $T_{sat}$, vapor surface tension $\sigma_{st}$, and latent heat of vaporization $i_{lv}$.

**Figure 2:**
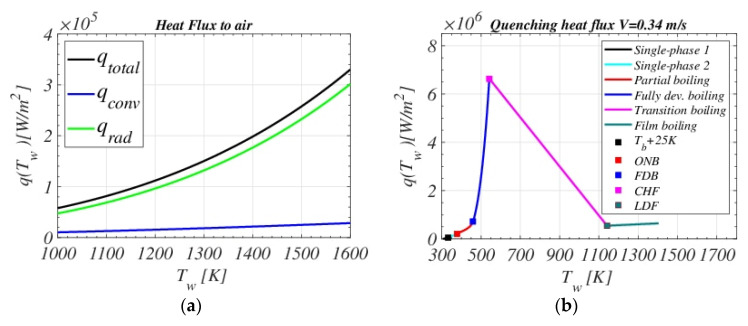
q vs. $T_{w}$ given by the thermal model when applied to the analyzed spindle. (**a**): heat flux during air transportation. (**b**): heat flux inside the quenching bath for V=0.34 m/s and $T_{b}$ = 35 °C.

The obtained heat flux function $q\left(T_{w}\right)$, which is explained in detail in the following Section 3.1.1, Section 3.1.2, Section 3.1.3, Section 3.1.4, Section 3.1.5 and Section 3.1.6, was implemented in MATLAB (https://www.mathworks.com/ (accessed on 25 May 2023)) Figure 2 shows the resulting dependency of the piece surface heat flux on its temperature $T_{w}$ for specific values of the agitation velocity V=0.34 m/s and the bulk temperature of the quenching bath $T_{b}=$ 35 °C. Figure 2a describes the air transportation stage, while Figure 2b shows q when the piece is submerged in the fluid tank.

**Figure 3:**
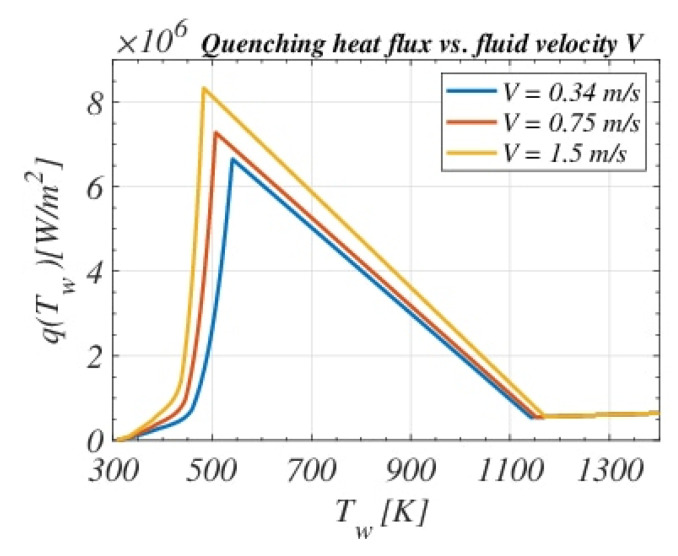
Heat flux dependency on different bath agitation velocities V for $T_{b}=35^{\;\circ }C$.

Each color represents one type of mechanism or heat flux at a specific regime. LDF, CHF, FDB (end of fully developed boiling regime), and ONB (onset of nucleate boiling) points are also highlighted. Figure 3 shows the surface heat flux dependency on the fluid velocity V for a quenching bath temperature $T_{b}=$ 35 °C. The Leidenfrost point LDF moves to higher temperatures when increasing the agitation velocity, which is consistent with the fact that the bigger the velocity, the sooner the destabilization of the vapor film occurs. In addition, for more intense agitations, $q_{CHF}$ is higher; that is, it enhances the nucleate boiling regime and shifts it to smaller temperatures. The following Section 3.1.1, Section 3.1.2, Section 3.1.3, Section 3.1.4, Section 3.1.5 and Section 3.1.6 describe in detail the functional $q\left(T_{w}\right)$ definition. 

#### 3.1.1. Heat Flux in Surrounding Air

During this stage, two different heat transfer phenomena occur. Firstly, the free convection to surrounding air (at a reference temperature $T_{ref}=$ 298 K) is modeled by a heat transfer coefficient $h_{c}=\frac{k_{air}{\bar{Nu}}_{cyl}}{L}$ (where the thermal conductivity of the air is denoted as $k_{air}$ and L refers to the characteristic piece length), which uses a free convection correlation for vertical cylinders extracted from [25]:

$${\bar{Nu}}_{cyl}={\left(0.825+\frac{0.387Ra_{L}^{\frac{1}{6}}}{{\left[1+{\left(\frac{0.492}{Pr}\right)}^{\frac{9}{16}}\right]}^{\frac{8}{27}}}\right)}^{2}\left(1+1.3\zeta^{0.9}\right)$$

$Ra_{L}$ and Pr, respectively, represent the Rayleigh and Prandtl dimensionless numbers of air, ζ is a curvature correction factor; and D is the spindle diameter (using an averaged value).

The radiative heat flux, $q_{rad}$, will be modeled by a common Stefan-Boltzmann expression, where the steel emissivity ϵ will follow a nonlinear dependency on the piece surface temperature $T_{w}$, as defined in [26]. This regime lasts until the piece is completely submerged in the fluid tank.

#### 3.1.2. Film Boiling

As in this stage the piece is at a very high temperature, radiative phenomena are still relevant, so that, a heat transfer coefficient h, which includes this effect, is proposed ([11,27]):

(4) $$h=h_{c}\left(1+0.025\left(T_{sat}-T_{b}\right)\right)+0.75h_{rad}$$

where $T_{sat}$ is the saturation temperature (373 K for pure water) and $T_{b}$, set as a constant value, is the bulk temperature of the quenching bath, measured far enough from the solid surface. The reference temperature will be $T_{ref}=T_{sat}$. Heat transfer coefficients $h_{rad}$ and $h_{c}$ stand for radiation and convection mechanisms, respectively. The first one, following [11], is expressed as

$$h_{rad}=\frac{\sigma_{SB}\left(T_{w}^{4}-T_{sat}^{4}\right)}{\left(\frac{1}{\epsilon_{w}}+\frac{1}{\epsilon_{l}}-1\right)\left(T_{w}-T_{sat}\right)}$$

where $\epsilon_{l}$ *y* $\epsilon_{w}$ are the fluid and the piece emissivities, and $\sigma_{SB}$ is the Stefan-Boltzmann constant. The convection coefficient is taken from [27]:

(5) $$h_{c}=0.94{\left[\frac{k_{v}^{3}\rho_{v}\left(\rho_{l}-\rho_{v}\right){i^{\prime }}_{lv}g}{L_{c}\mu_{v}\left(T_{w}-T_{sat}\right)}\right]}^{1/4}$$

The vapor properties are denoted, using the subscript v, as $\mu_{v}$, $k_{v},$ and $\rho_{v}$ (viscosity, conductivity, and density, respectively). The characteristic length of the film effects is defined as $L_{c}=\sqrt{\frac{\sigma_{st}}{g\left(\rho_{l}-\rho_{v}\right)}}$, being $\sigma_{st}$ the surface tension between the liquid and the vapor, g is the gravity acceleration, and $\rho_{l}$ is the liquid density. A modified latent heat, used to model the energy invested in heating the liquid from $T_{b}$ until evaporation temperature and then vaporizing it, is defined and named as ${i^{\prime }}_{lv}$.

(6) $${i^{\prime }}_{lv}=C_{p,l}\left(T_{sat}-T_{b}\right)+i_{lv}+C_{p,v}\left(T_{w}-T_{sat}\right)$$

$C_{p,v}$ and $C_{p,l}$ are the fluid specific heat of the vapor and liquid phases, respectively, and $i_{lv}$ is the latent heat of evaporation.

In Equations (5) and (6), vapor properties are taken at $T_{sat}$ and liquid properties are taken at $T_{b}$.

#### 3.1.3. Transition Boiling

The vapor blanket starts to destabilize at the Leidenfrost temperature $T_{LDF}$, calculated using the expression proposed by [28]:

(7) $$T_{LDF}=\left(550+50\;\sqrt{V+V_{flot}}+3\left(T_{sat}-T_{b}\right)\right)\;C_{a}$$

In solving the film boiling Equation (4) at a wall temperature equal to that Leidenfrost temperature ($T_{w}=T_{LDF}$), the heat flux $q_{LDF}$ is obtained. Previous laboratory quenching experiments [26] have been used to adjust the coefficient defined as $C_{a}=1.6$ in Equation (7). The characteristic liquid flux velocity of the quenching bath (increased by the immersion velocity in the first seconds) is referenced as V, while $V_{flot}$ represents the buoyancy-induced velocity associated with the presence of vapor bubbles in the liquid:

(8) $$V_{flot}=\sqrt{\frac{\rho_{l}-\rho_{v}}{\rho_{l}}gL}$$

Again, the liquid density $\rho_{l}$ in Equation (8) is evaluated at saturation temperature $T_{sat}$.The heat flux dependency on the surface piece temperature $T_{w}$ in this region has been assumed to be linear:

$$q=q_{LDF}-\left(\frac{q_{LDF}-q_{CHF}}{T_{LDF}-T_{CHF}}\right)\left(T_{LDF}-\left(T_{w}-T_{sat}\right)\right)$$

The maximum value of the heat flux $q_{CHF}$ and its corresponding temperature $T_{CHF}$ are detailed in the following subsection.

#### 3.1.4. Fully Developed Boiling

This stage starts once the maximum value of the heat flux, named Critical Heat Flux and referenced as $q_{CHF}$, is reached [29],

(9) $$q_{CHF}=71987\;\sqrt{V+V_{flot}}\left(T_{sat}-T_{b}\right)$$

Then, the heat flux is reduced as the surface piece temperature decreases [30]:

(10) $$q={\left[1058h_{l}\left(T_{w}-T_{sat}\right){\left(\rho_{l}\left(V+V_{flot}\right)i_{lv}\right)}^{-0.7}\right]}^{3.33}$$

being $h_{l}$ a single-phase heat transfer coefficient, defined in Equation (11) by the Dittus-Boelter correlation [31]:

(11) $$h_{l}=0.0243Re_{l}^{0.8}Pr_{l}^{0.4}\left(k_{l}/e\right)$$

where the bulk temperature $T_{b}$ is used to evaluate the dimensionless numbers $Re_{l}$ and $Pr_{l}$ (the Reynolds and Prandtl numbers of the liquid). The Reynolds number is based on the previously defined fluid velocity V and the characteristic dimension perpendicular to the fluid flow, e. As the pieces remain in vertical position (as can be seen in Figure 1), the averaged piece thickness e was taken as the characteristic length in Equation (11). $T_{CHF}$ is calculated by solving the temperature value in Equation (10) for the heat flux obtained in Equation (9): $q=q_{CHF}$.

#### 3.1.5. Partial Boiling

In this regime, as the vapor bubble nucleation phenomenon decreases in intensity, the contribution of the nucleate boiling becomes of the same order as the monophasic forced convection heat flux. As a result, a smooth transition between these two regimes previously characterized by (10) and (11) is sought. As in [32], two points, named fully developed boiling (FDB) and onset of nucleate boiling (ONB), delimit this transition stage: The estimation of the first point follows $q_{FDB}=1.4q_{D}$ where the heat flux $q_{D}$ is the intersection of the Dittus–Boelter correlation (11) with the fully developed boiling Equation (10), and is solved iteratively in Equation (12):

(12) $$q_{D}={\left[1058\left(q_{D}-h_{l}\left(T_{sat}-T_{b}\right)\right){\left(\rho_{l}\left(V+V_{flot}\right)i_{lv}\right)}^{-0.7}\right]}^{3.33}$$

$T_{FDB}$ can be obtained using Equation (10) and substituting the previously calculated value $q_{FDB}$. The end of the partial boiling regime is set by the temperature $T_{ONB}$, which is obtained as follows ([32]):

$$T_{ONB}=T_{sat}+\frac{4\sigma_{st}T_{sat}h_{l}}{i_{lv}k_{l}\rho_{v}}\left[1+\sqrt{1+\frac{k_{l}i_{lv}\left(T_{sat}-T_{b}\right)\rho_{v}}{2\sigma_{st}T_{sat}h_{l}}}\right]$$

where liquid properties are evaluated at $T_{b}$. The heat flux value at the ONB point ($q_{ONB}$) is calculated using the Dittus–Boelter single-phase correlation of Equation (11) for $T_{ONB}$.

Finally, partial boiling heat flux is modeled according to [32] using the following equation.

$$q=q_{ONB}+\frac{q_{FDB}-q_{ONB}}{{\left(T_{FDB}-T_{sat}\right)}^{m}-{\left(T_{ONB}-T_{sat}\right)}^{m}}\cdot \left({\left(T_{w}-T_{sat}\right)}^{m}-{\left(T_{ONB}-T_{sat}\right)}^{m}\right)$$

where m is obtained for each point following $m=1+\frac{2.33}{q_{FDB}-q_{ONB}}\left(q-q_{ONB}\right)$, starting with m=3.33 at $T_{FDB}$ and ending with m=1 at $T_{ONB}$.

#### 3.1.6. Single-Phase Heat Flux

The last regime, where the vapor bubbles have completely disappeared, is characterized by forced convection between the piece and the monophasic fluid. The single-phase correlation (11) is used ($q=h_{l}\left(T_{w}-T_{b}\right)$) for wall temperatures below $T_{ONB}$. However, for temperatures smaller than $T_{b}+25\;K$, Dittus–Boelter’s (Equation (11)) significantly over-predicts the heat flux values. To adjust a new convective heat transfer equation sensitive to fluid velocity and capable of retaining the variation of the liquid viscosity with temperature, CFD techniques and a least-squares adjustment have been used [24], obtaining the following expression:

$$h_{l1}=0.097Re_{l}^{0.612}Pr_{l}^{0.23}{\left(\frac{\mu_{l}}{\mu_{sat}}\right)}^{0.175}\left(\frac{k_{l}}{e}\right)$$

### 3.2. Validation of the Thermal Model

**Figure 4:**
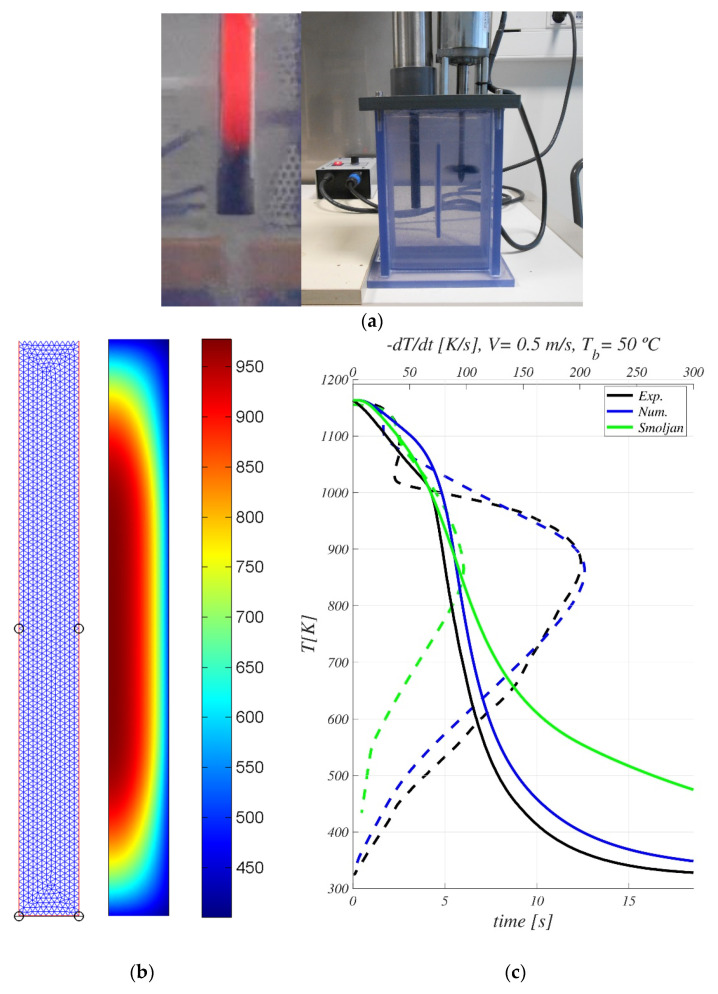
(**a**): Picture of the experimental Inconel 600 standard probe tested (**left**) and the experimental quenching container with agitation (**right**). (**b**) Computational mesh and temperature field in the probe for Test #5 at t = 5 s. (**c**): Evolution of temperature T vs. time t (solid lines) and cooling rate dT/dt vs. T (dashed lines) at the center of the standard experimental probe for Test #5.

The surface heat flux function $q(T_{w})$ described in Section 3.1 was solved using the software Matlab and then imported as an external tabular wall temperature-dependent function in the software COMSOL-Multiphysics (version V 3.5a), where the complete thermal model (1)–(3) was implemented (see description in Section 3.5), solved, and validated by comparison with experiments on a standard lab probe, as the one shown in Figure 4a.

According to the international standard ISO 9950 and the American standards ASTM D 6200-01 and ASTM D 6482-99, cylindrical test probes (12.5 mm in diameter and 60 mm in length) have been used. A data acquisition unit connected to a k–type thermocouple, located at the center of the probe, has been used to save temperature data every 0.01 s. Since it is intended to validate the thermal model, the alloy Inconel 600, which does not undergo any metallurgical changes when cooled, is used to fabricate the test probes. The quenching container, of section 125 mm × 60 mm and height 205 mm, was provided by Swerea/IVF (https://www.ri.se/en/what-we-do/services/ivf-smartquench-for-control-of-cooling-curve-measurement (accessed on 25 May 2023)) and filled with 1.2 L of water. The container has an agitation device whose velocity can be set from 0 to a maximum of 1.2 m/s. The probe was heated in an electric furnace until a uniform temperature $T_{0}=1163$ K was reached. To assure homogenization, the piece remained inside the furnace at the objective temperature for at least 5 min. Once heated, the piece was submerged in the experimental container in less than 1.5 s. Five different experimental tests were carried out with three agitation velocities, V= 0.34, V= 0.5, and V= 0.75 m/s, and different bulk fluid temperatures, $T_{b}=20^{\;\circ }C$, $T_{b}=35^{\;\circ }C$ and $T_{b}=50^{\;\circ }C$, as indicated in Table 2.

For these test conditions, the thermal model (1)–(3) with Q=0 was solved in a 2D-axisymmetric domain (of dimensions equal to the radius and length of the cylinder probe) using the unstructured mesh of Figure 4b formed by 2636 triangles. The initial uniform piece temperature and fluid bulk temperature were selected to be equal to those of the experiment. In these cases, heat transfer during the transportation of the fluid was neglected. A sensitivity analysis for the simulations with the standard probe was carried out but omitted for the sake of brevity. The computational time for each case (processed in serial) took 20 min on a workstation with 128 GB of RAM equipped with Intel-Xeon processors. As an example, Figure 4b shows the snapshot of the probe temperature field at t = 5 s for Test #5.

Table 2 shows the relative deviations between the numerical results and the experimental measurements for the five tests: averaged and maximum relative deviations in temperature, relative deviations on the predicted maximum cooling rate, and on their predicted temperature $T_{CHF}$. Averaged deviations between the numerical prediction and the measured temperatures remain lower than 7.5% for all the tests. An example of the comparison between the numerical and experimental thermal evolutions is shown in Figure 4b for Test #5. Solid lines correspond to the cooling curve at the center of the probe, $T_{c}$ vs. time t, while dashed lines represent the cooling rate at the center of the probe vs. its temperature $T_{c}$. Black lines show the experimental measurements, while the numerical predictions are plotted in blue.

To illustrate the accuracy of the thermal model with the experimental results when compared with other simplified thermal models based on correlations extensively used in the microstructure prediction in quenching processes, problems (1)–(3) have been numerically integrated assuming the correlation approach of Smoljan [14], which approximates the heat flux at the piece surface (Equation (2)) by a triangular function. The green lines in Figure 4b show these numerical results for Test #5, where poor agreement with the experimental measurements is observed. This behavior is consistently obtained for all tests. Therefore, despite the simplicity of the thermal model proposed in Section 3.1, its numerical results provide a huge improvement in accuracy when compared to the common approaches used to predict the thermal history and, therefore, the final microstructure of the piece after treatment.

### 3.3. Metallurgical Model

During the cooling process, the evolution of the different micro-structural phases of the steel should be determined and coupled to the thermal problem described in Section 3.1. The transformations dominated by carbon diffusion processes (that potentially lead to ferrite f, pearlite p, and bainite b phases from the austenite γ phase) have been modeled by an Avrami-type equation as described in [24].

$$X_{i}=X_{\gamma }X_{i,max}\left[1-exp\left(-b{\left(\frac{d_{\gamma }}{d_{\gamma ,ref}}\right)}^{m}t^{n}\right)\right]$$

where the proportion of each microconstituent i is named as $X_{i}$, $X_{i,max}$ is the maximum proportion of microconstituent i at a given temperature, and $X_{\gamma }$ is the proportion of austenite at the beginning of the transformation. In addition, the model uses two material parameters, which are extracted from the TTT diagram: b and n. As mentioned, the composition of the industrial steel is quite similar to the 25 M 6 Steel, so its TTT diagram, extracted from [33], was used. Parameter $\frac{d_{\gamma }}{d_{\gamma ,ref}}$ stands for the fraction between the piece’s austenitic grain size (taken in this study as 6.5 ASTM) and the reference austenitic grain size used in the TTT diagram. The discretization of the cooling in small intervals of constant temperatures has been done by applying the additive rule of Scheil [24], used for continuous cooling. The following expression shows the criterion that determines the beginning of transformation:

$$\sum \frac{\Delta t_{i}}{\tau_{i}\left(\frac{d_{\gamma }}{d_{\gamma ,ref}}\right)}\geq 1$$

where $\tau_{i}$ is the incubation time of the microconstituent i for each temperature, given by the TTT diagram.

The martensite proportion $X_{m}$ is modeled according to the Koïstinen–Marburger time-independent algebraic Equation (13) (see [24]).

(13) $$X_{m}=\left(1-X_{f}-X_{p}-X_{b}\right)\left\{1-exp\left(\beta {\left[M_{s}-T\right]}^{+}\right)\right\}$$

where the temperature for the beginning of martensitic transformation ($M_{s}$) is extracted from the CCT (Continuous Cooling Transformation) diagram of the 25 M 6 Steel. $X_{f}$, $X_{p}$ y $X_{b}$ are the ferrite, pearlite, and bainite proportions, and β is a material parameter.

### 3.4. Mechanical Model

The mechanical model provides the residual stresses and final deformations of the piece induced by the heat treatment. The quasi-static and small deformations mathematical model used relates the stress tensor σ and the strain tensor ε, the latter being:

$$\varepsilon =\varepsilon^{e}+\varepsilon^{p}+\varepsilon^{th}+\varepsilon^{pt}$$

where $\varepsilon^{e}$, $\varepsilon^{p}$, $\varepsilon^{th}$, and $\varepsilon^{pt}$ are the elastic, plastic, thermal, and transformation-induced plasticity (TRIP) contributions. The elasticity problem was characterized by an isotropic Young’s modulus E and Poisson coefficient ν that depend on the temperature of the material [20]. The Von Mises criteria have been used to define the fluence function F that determines the plasticity region ($F\left(\sigma \right)=0$):

$$F\left(\sigma \right)=\sigma_{eq}-\sigma_{y}\left(T,X\right)-R\left(T,X,r\right)=0$$

where T represents the temperature, X the microstructure proportion (in vectorial form), $\sigma_{eq}$ the Von Mises stress, $\sigma_{y}$ and R the yield stress, and the hardening law for the multiphasic material (both dependent on the temperature and the microstructure). Thus, the strain plastic tensor will be defined as

$${\dot{\varepsilon }}_{ij}^{p}=\dot{\lambda }\frac{\partial F}{\partial \sigma_{ij}}$$

where $\dot{\lambda }$ stands for the plastic multiplier. Thermal strains, which include deformations associated with the volumetric change generated by the metallurgical transformations, are described by the following Equation ([34]):

$$\begin{array}{ccc} \varepsilon^{th} & = & \left(1-X_{f}-X_{p}-X_{b}-X_{m}\right)[\alpha_{\gamma }\left(T\right)\left(T-T_{ref}\right)I-\\ & - & \left(\varepsilon_{f}^{th}\left(T_{ref}\right)-\varepsilon_{\gamma }^{th}\left(T_{ref}\right)\right)I]+\\ & + & \left(X_{f}-X_{p}-X_{b}-X_{m}\right)\left[\alpha_{f}\left(T\right)\left(T-T_{ref}\right)I\right] \end{array}$$

where $\alpha_{f}$ is the thermal dilatation coefficient for the microconstituents ferrite, pearlite, bainite, and martensite, while $\alpha_{\gamma }$ stands for austenite. $T_{ref}$ is a reference temperature for the cold phases (the reference state), where the term $\varepsilon_{f}^{th}\left(T_{ref}\right)-\varepsilon_{\gamma }^{th}\left(T_{ref}\right)$ depicts the difference in compactness between the crystallographic structures at the reference temperature.

Finally, the TRIP deformation is modeled with Equation (14) as in [35], which involves the stress state and the evolution rate of the transformations:

(14) $${\dot{\varepsilon }}^{pt}=\frac{3}{2}\sigma^{D}\sum_{i=1}^{4}K_{i}{F_{i}}^{\prime }\left(1-X_{\gamma }\right)\langle {\dot{X}}_{i}\rangle$$

being $\sigma^{D}$ the deviatoric stress tensor, $X_{\gamma }$ the austenite proportion, $\left\langle {\dot{X}}_{i}\right\rangle$ the positive value of the velocity rate of the cold microconstituents, $K_{i}$ characteristic constants associated to each phase i, and ${F_{i}}^{\prime }$ representing the derivatives of a normalized function $F_{i}$ that fulfills $F_{i}\left(0\right)=0$ and $F_{i}\left(1\right)=1$. These last two terms are characteristic of each material [35].

### 3.5. Numerical Implementation

**Figure 5:**
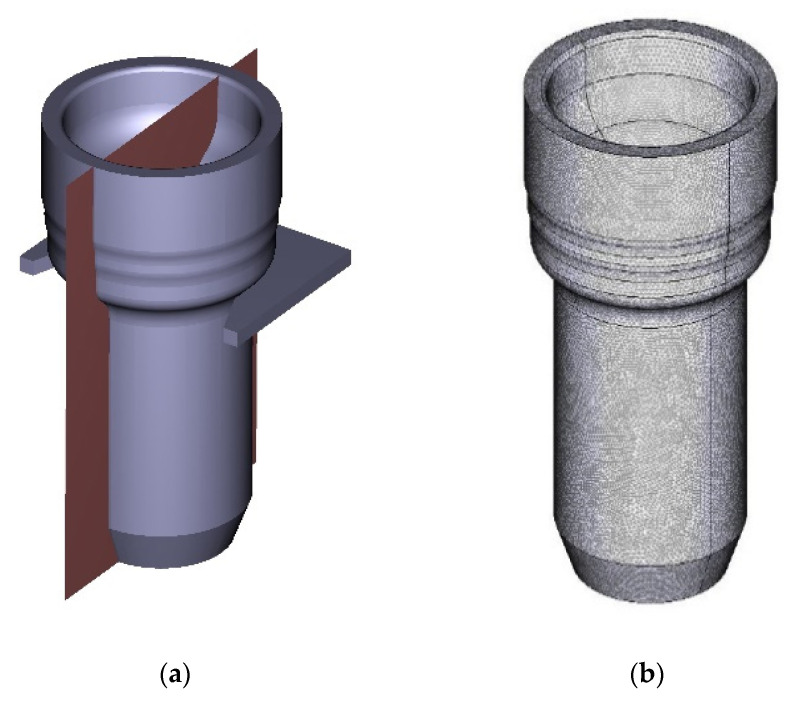
(**a**): Sketch of the spindle geometry and part of the supporting device. (**b**): Computational mesh.

The complete thermal-metallurgical-mechanical model described in Section 3 has been numerically solved for a 3D computational domain of a complete spindle. As each piece is placed on a supporting device, the corresponding contact surface with the tray is differentiated in the thermal–metallurgical model of the spindle, assuming a constant heat transfer coefficient of ([36]). In addition, at the contact boundary between the supporting device and the spindle, the displacements of the piece were blocked. A uniform temperature of $T_{0}=1163$ K, which corresponds to the homogenizing furnace temperature, was considered the initial condition. Spindle geometry and mesh, formed by 391,530 hexahedrons, can be seen in Figure 5b, while part of the supporting tray and the vertical symmetry plane, which divides the piece and the supporting device, are depicted in Figure 5a.

**Figure 6:**
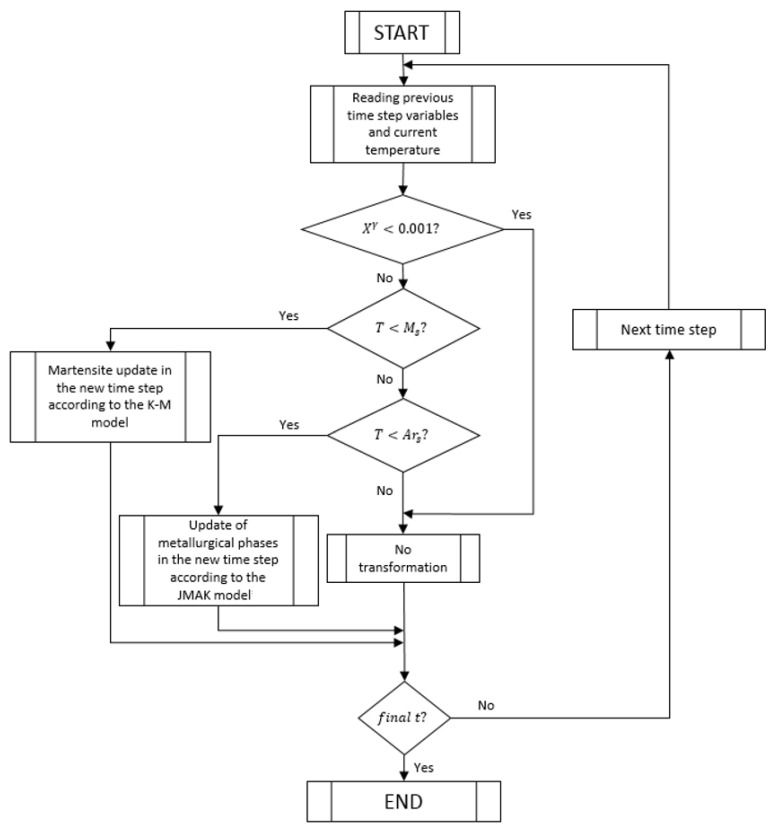
Metallurgical model resolution algorithm.

**Figure 7:**
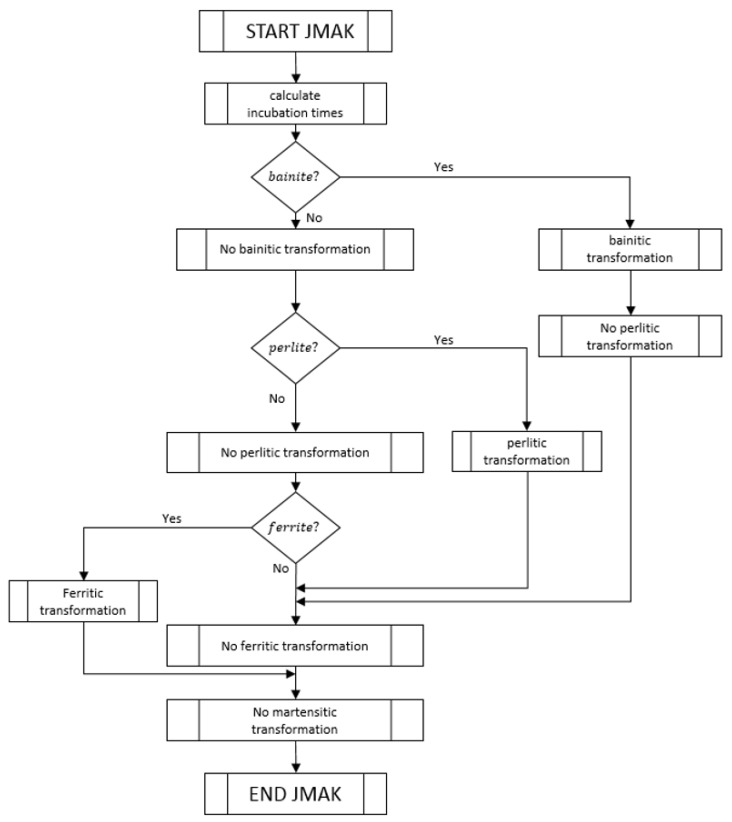
JMAK model resolution algorithm (referenced in the metallurgical model resolution algorithm described in Figure 6).

The metallurgical equations, described in Section 3.3, have been programmed in the software Matlab (version V 11)following the resolution algorithm shown in the block diagrams of Figure 6 and Figure 7. The complete thermal–metallurgical model described in Section 3.1 and Section 3.3 has been programmed using finite element methods (FEM) in Matlab, using its connection with Comsol Multiphysics v3.5a (https://www.comsol.com/, accessed on 25 May 2023) (check [37] for more information on Comsol-Matlab LiveLink). First-order elements P1 were used, linear systems were solved by the generalized minimal residual (GMRES) method, and a backward differentiation formula (BDF) scheme (of order 5) was used for time integration. Once the piece temperature and microstructure evolutions have been determined, these evolutions are exported and introduced as inputs in the mechanical model (defined in Section 3.4). Free software Code-Aster v11.6, developed by Electricité de France (Electricité de France. http://www.code-aster.org (accessed on 25 May 2023)) has been used to integrate and solve this mechanical problem.

**Figure 8:**
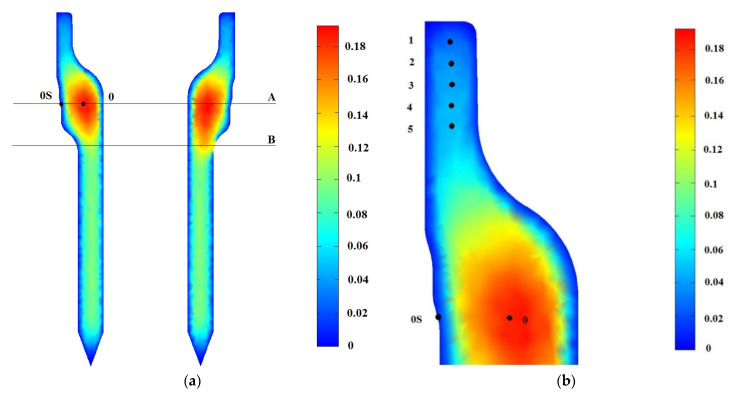
(**a**): Final bainite content along the symmetry plane that divides the piece and the supporting device. (**b**): Detail of the upper part of the spindle.

A sensitivity analysis of the results was carried out for three different meshes and three step sizes for the thermal–metallurgical model. Table 3 shows the relative errors of the maximum cooling velocity $\varepsilon_{1}$ (evaluated at point 0*S*, indicated in Figure 8) and of the maximum final bainite content in the piece $\varepsilon_{2}$ (evaluated at point 0 of Figure 8). The final selected mesh was Mesh 2# of Table 3 with the corresponding step size of Δt= 0.025 s, while for the mechanical problem, the use of a step size of 1 s was sufficient.

A workstation with 128 GB of RAM and two Intel-Xeon processors (6 nodes and 1.8 GHz) was used. The computational times were 50 h for the thermal–metallurgical model (run in serial) and 10 h for the mechanical model. The difference is justified because the thermal–metallurgical model needs a finer temporal discretization to retain the high temperature gradients that appear in the first seconds of the quenching.

## 4. Results

Figure 1a shows the final bainite content along the vertical symmetry plane that divides the piece and the supporting device. The right part corresponds to the section where the piece is in contact with the tray. The corresponding final martensite content is $1-X_{b}$. The maximum baitine content is obtained in the thickest region of the piece. Table 4 shows the bainite content given by the numerical model and by the (averaged) metallurgical analysis of 40 pieces from different steel casts (provided by the company) at specific locations indicated in Figure 8: point 0 (piece core center, belonging to line A), and point 0*S* (piece surface).

**Figure 9:**
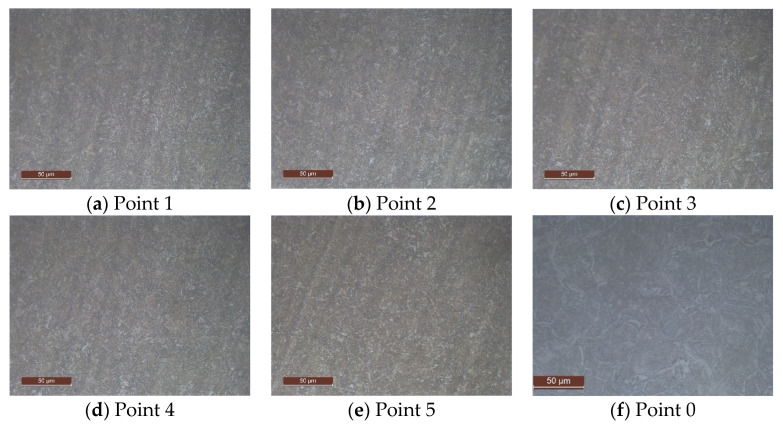
Micrographs (500×) corresponding to the marked points 1 to 5 and 0 taken from probes of a spindle subjected to the described industrial quenching process. Ruler in micrographs indicated 50 μm.

Micrographs corresponding to specific points of a spindle subjected to the industrial quenching process are shown in Figure 9. Micrographs were obtained by applying a 2% Nital etch solution to the piece samples and obtained at 500× magnification by using an optical metallurgical microscope, the Olympux GX 51. The analyzed points 1 to 5, indicated in Figure 6b, correspond to depths equal to 5 mm, 10 mm, 15 mm, 20 mm, and 25 mm from the top of the spindle, respectively. At points 1 to 5, only martensite (with residual bainite) and no ferrite are encountered, while at point 0, an average content of bainite of 0.15 is found.

**Figure 10:**
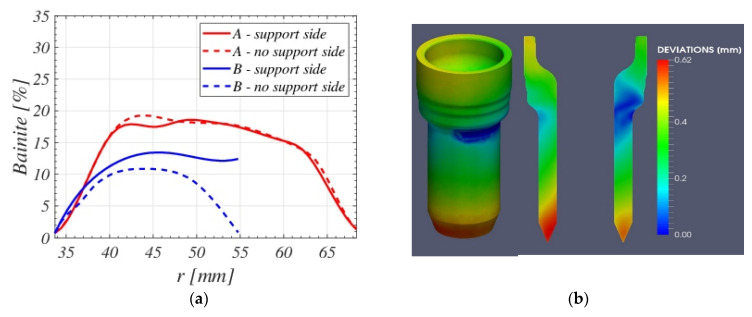
(**a**): Bainite content along lines A (red) and B (blue) of Figure 6. Solid lines correspond to the support side. (**b**): Numerical deviations (in mm) predicted by the model after the quenching treatment.

Special attention should be given to the cold regions created by the contact of the pieces with the supporting device. Figure 10a shows the final bainite content across lines A and B for the two sections of Figure 8a. As the right section is in contact with the supporting device, the corresponding content across line B is different from its counterpart in the left section. These differences are entirely created by the different cooling rates that the two sections experiment with, which generate different final micro-constituent structures and deformations.

### 4.1. Direct Quenching Prediction

In order to test the capabilities of the model to predict the final piece microstructure and deformations, the complete model has been solved to predict the quenching under different process conditions. Instead of the industrial quenching process in batches, described in Section 2, a direct quenching was considered, that is, a quenching of the pieces just after the forging, therefore eliminating the heating step in the homogenization furnace. Provided that the piece has the required final properties, this change in the industrial process would be profitable in terms of reducing consumption of natural gas, emissions of contaminants, and costs. The company made some direct quenching tests, which were used to validate the numerical model. For one test campaign, the initial temperature of the rods before forging, which was considered uniform, was equal to 1358 K. After the transportation of the rods and the forging in die-punches, the pieces (one by one) were transported with the supporting device from the forging area and submerged in the stirred water tank. Transportation time after forging and before submerging was 90 s in the tests. Thus, as the temperature of the piece after transportation time has fallen substantially, ferrite micro-constituents are expected to appear in these quenching conditions. The non-uniform temperature of the spindle just after the forging process, needed as an initial condition for the quenching model, was imported from a ${FORGE}^{®}$ (http://www.transvalor.com (accessed on 25 May 2023)) (Forge NxT 2.1, Transvalor, Nice, France) simulation. This simulation was provided by the company and was obtained from a thermos-mechanical model solved to mimic the complete forging stage (check [38,39] to see similar problems solved with this software). The temperature field was then projected over the spindle mesh described previously and used as the initial condition $T=T_{0}\left(\vec{x}\right)$ for the complete quenching numerical model. The rest of the quenching process parameters (fluid velocity V and temperature $T_{b}$) were the same as for the industrial quenching process except for the austenitic grain size (7 ASTM), as the homogenization furnace is not present in the direct quenching.

**Figure 11:**
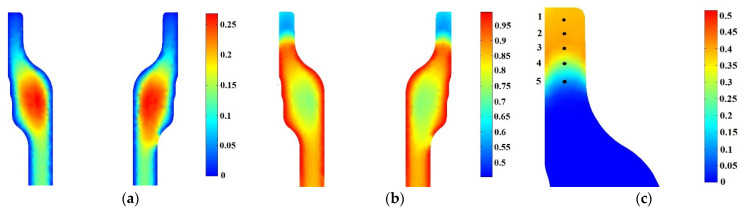
Prediction of final content of (**a**) bainite, (**b**) martensite and (**c**) ferrite for direct quenching.

**Figure 12:**
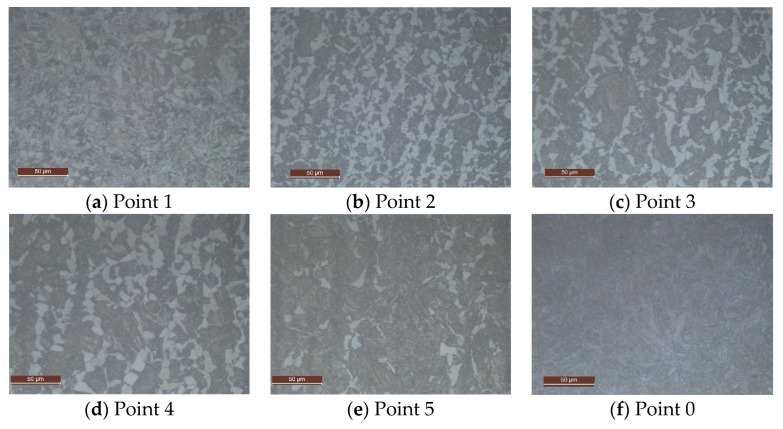
Micrographs (500×) corresponding to the marked points 1 to 5 and 0 taken from probes of a spindle subjected to the alternative direct quenching process.

Figure 11 shows, respectively, the bainite $X_{b}$, martensite $X_{m}$, and ferrite $X_{f}=1-X_{b}-X_{m}$ final proportion predicted by the model for the vertical symmetry plane. A comparison with experimental data extracted from micrographic analyses at points 0 to 5 across a spindle section, shown in Figure 12, was done. Different ferrite proportions were found at points 1 to 5. Figure 12f shows the micrograph at point 0, which corresponds to a bainitic–martensitic structure.

Table 5 shows deviations between the measured and predicted ferrite proportions at locations 1 to 5 and 0 of the piece. Additionally, it includes the experimental measurements of the Vickers hardness HV at those specific points of the spindle after the subsequent tempering heat treatment (of approximately 2 h at 540 °C). 

**Figure 13:**
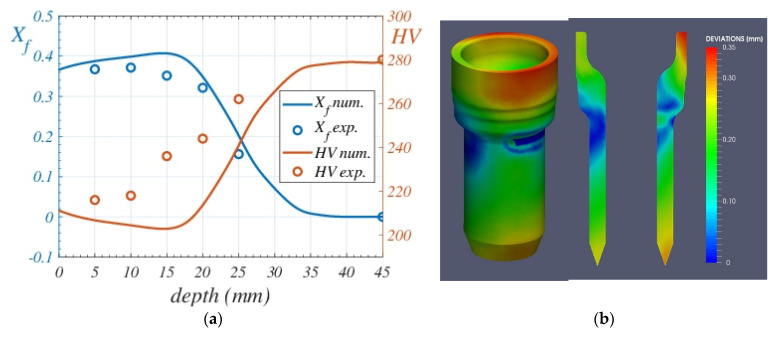
(**a**): Ferrite content along the depth of the piece: experimental values (blue dots) and numerical prediction (blue line). HV hardness: experimental measurements (red dots) and numerical (red line). (**b**): Numerical deviations (in mm) predicted by the model after the direct quenching process conditions.

Numerical predictions of the final micro-constituents after the direct quenching treatment follow the trends of the experimental data along the depth of the piece, providing a mean relative error of less than 12.1% for the evaluated points, therefore proving the capability of the model to predict the final microstructure composition of the piece under different quenching conditions. Figure 13a shows again the experimental (blue dots) and the numerical prediction (blue line) of ferrite content along the depth of the piece, as well as the spindle HV hardness measurements after tempering (red dots) and the numerical hardness prediction (red line). The numerical hardness has been obtained using the formulas developed in [40]. A value of HV 270 was assumed for a pure martensitic structure after tempering.

Geometrical deviations are shown in Figure 13b, where the pattern is completely different from the usual industrial quenching process in batches with a homogenization furnace. Unfortunately, comparisons of geometrical deviations could not be made as the treated spindles were discarded by the company.

## 5. Conclusions

A thermal–metallurgical and mechanical model to predict the evolution of the microstructure and mechanical properties of steel automotive spindles subjected to a quenching heat treatment is presented. The model is based on an innovative heat transfer characterization capable of retaining the different regimes that appear in the real process, which depend explicitly on the thermophysical properties of the quenching fluid, its bulk temperature, its velocity upstream of the piece, and the characteristic dimensions of the piece.

The validation of the thermal model shows averaged deviations in the temperature prediction of less than 7.5%, which means an improvement in accuracy of 42% with respect to the common approaches used in this type of industrial problem.

The complete (thermal–metallurgical and mechanical) model has been tested for two different quenching processes of steel automotive spindles in a company: (i) a batch-type industrial quenching process where pieces after forging are subjected to homogenization heating before immersion in a water tank; and (ii) a direct quenching process where each piece is submerged directly in the fluid just after being forged.

Comparison of metallurgical results shows very good agreement with the industrial results, with averaged deviations for the tested data of 10% for the standard industrial process and of 12.1% for the direct quenching tested.

All in all, the following conclusions can be extracted from this study:

- A prediction tool has been developed for the design of the industrial quenching process.
- The computational cost-accuracy relationship has been optimized with respect to the models proposed in the literature for this type of process.
- The appearance of unwanted microstructures can be predicted by the model with good accuracy.
- The proposed model allows us to find out the mechanical properties of the product based on the composition of the steel used and the process parameters. Innumerable plant tests required for the adjustment of process parameters can be avoided by using this tool.
- The proposed model can be used to redesign and optimize the quenching process, for example, to reduce energy consumption, e.g., by adjusting the homogenization temperature, minimizing operating times, or even eliminating the homogenization step (direct quenching), if process times and parameters are adjusted to avoid the appearance of ferrite.

## Figures and Tables

**Table 1:** Experimental piece superficial temperatures *T_w_* at different times during the transportation to the quenching bath.

Test	*T_w_*_1_ [K]	*T_w_*_2_ [K]	*T_w_*_3_ [K]	*T_w_*_4_ [K]
1	1082	1073	1063	1062
2	1093	1093	1078	1068

**Table 2:** Deviations (averaged and maximum temperature deviations, maximum cooling rate, and $T_{CHF}$ deviations) between experimental measurements and numerical results of the thermal problem for the cylindrical probe.

Test	*V* (m/s)	*T_b_* (°C)	Aver. (%)	Max (%)	Max. Cooling Rate (%)	*T_CHF_* (%)
#1	0.34	35	4.0	11.2	5.2	9.3
#2	0.5	35	3.9	12.4	1.8	6.2
#3	0.75	35	4.6	16	8.6	3.4
#4	0.5	20	4.8	15.6	6.9	4.2
#5	0.5	50	7.5	13	1.6	1.9

**Table 3:** Sensitivity analysis of the numerical problem.

**Mesh**	**Nº of elements**	**∆** ***t*[** ***s*]**	***ε*_1_[%]**	***ε*** **_2_[%]**
1#	264664	0.025	4.29	0.71
2#	391530	0.025	1.38	0.93
3#	607746	0.025	*−−*	*−−*
**Mesh**	**Nº of elements**	**∆** ***t*[*s*]**	***ε*_1_[%]**	***ε*** **_2_[%]**
2#	391530	0.1	1.4	1.36
2#	391530	0.025	0.13	0.13
2#	391530	0.01	*−−*	*−−*

**Table 4:** Comparison of the final bainite proportion at points 0 and 0*S* predicted by the numerical model and obtained from the metallurgical analysis of the treated spindles.

Point	*X_b_* Num.	*X_b_* Exp.	*ε_b_* [%]
0	0.18	0.15	20
0*S*	0	0	0

**Table 5:** Comparison of the final ferrite proportion at points 1–5 and 0 (Figure 9) given by the metallurgical analysis and the numerical model for the spindles subjected to direct quenching process.

Point	*X_f_* Exp.	*X_f_* Num.	*ε_f_* [%]	*HV* exp.
1	0.36	0.39	8.3	216
2	0.37	0.40	8.1	218
3	0.35	0.41	17.1	236
4	0.32	0.34	6.25	244
5	0.15	0.20	33	262
0	0	0	0	280

## Data Availability

Not applicable.

## Nomenclature

$C_{p}$	specific heat at constant pressure
D	averaged piece diameter
E	Young modulus
Gr	Grashof number $\frac{g\rho^{2}\beta L^{3}\left(T_{w}-T_{ref}\right)}{\mu^{2}}$
L	piece length
$L_{c}\;$	characteristic length $\sqrt{\frac{\sigma_{st}}{g\left(\rho_{l}-\rho_{v}\right)}}$
$M_{s}$	onset temperature for martensitic transformation
Pr	air Prandtl number $\frac{C_{p}\mu }{k}$
$Pr_{l}$	liquid Prandtl number
Q	heat flux per unit of volume generated by metallurgical transformations.
$Ra_{L}$	air Rayleigh number, $Gr_{L}Pr$, based on piece length L
$Re_{l}$	liquid Reynolds number $\frac{\rho eV}{\mu }$
T	temperature
$T_{b}$	liquid bulk temperature
$X_{i}$	proportion of the metallurgical phase i
R	hardening law for multiphasic materials
V	fluid velocity
$V_{flot}$	characteristic velocity induced by buoyancy $\sqrt{\frac{\rho_{l}-\rho_{v}}{\rho_{l}}gL}$
d	grain size
e	averaged piece thickness
g	gravity
h	heat transfer coefficient
$h_{l}$	single phase (liquid) heat transfer coefficient
$i_{lv}^{'}$	modified latent heat
$i_{lv}$	latent heat of evaporation
k	thermal conductivity
q	heat flux per unit of surface
t	time
**Greek symbols**	
α	solid thermal dilatation coefficient
ΔH	enthalpy of solid phase change
ϵ	emissivity
ε	strain tensor
$\dot{\lambda }$	plasticity multiplier
μ	viscosity
ν	Poisson coefficient
ρ	density
σ	stress tensor
$\sigma_{SB}$	Stefan-Boltzmann constant
$\sigma_{st}$	liquid-vapor surface tension
$\sigma_{eq}$	Von Mises stress
$\sigma_{y}$	yield stress
$\sigma^{D}$	deviatoric stress tensor
τ	incubation time for a micro-constituent
**Subscripts**	
CHF	critical heat flux
LDF	Leidenfrost point
FDB	fully developed boiling
ONB	onset of the nucleate boiling
c	convection
b	bainite
f	ferrite
γ	austenite
i	metallurgical phase index
l	liquid phase
m	martensite
p	pearlite
rad	radiation
sat	saturation
v	vapor phase
w	wall
